# Decision-making for pediatric vaccination scheduling under multiple constraints: a case study from China^✰^

**DOI:** 10.3389/fpubh.2026.1768367

**Published:** 2026-03-06

**Authors:** Xiaochen Ma, Qiang Huang, Shanshan Chai, Mingxia Zhang

**Affiliations:** 1 School of Economics and Management, Dongguan University of Technology, Dongguan, Guangdong, China; 2 Nanshan District Center for Disease Control and Prevention, Shenzhen, Guangdong, China; 3 College of Management, Shenzhen University, Shenzhen, China

**Keywords:** balanced scheduling, influenza outbreak, optimization, pediatric vaccination, service capacity

## Abstract

**Introduction:**

Providing timely and equitable pediatric vaccination services is a fundamental responsibility of public health systems. As vaccination schedules become more complex-with an increasing number of vaccines, age-specific eligibility rules, and strict dose interval requirements-vaccination centers are under growing pressure to manage routine services while still being able to respond to epidemic-related demand.

**Methods:**

This study presents a quantitative decision-support approach designed to assist pediatric vaccination scheduling under real-world operational constraints, including age eligibility, inter-dose intervals, and daily service capacity. Using data from a public health vaccination center in Shenzhen, China, the approach is applied to develop balanced vaccination schedules for both routine childhood immunization and periods of increased demand during influenza outbreaks.

**Results:**

Compared with conventional walk-in or unscheduled vaccination practices, the proposed approach noticeably reduces day-to-day fluctuations in vaccination volume and makes service delivery more predictable. Daily vaccination workloads stabilize over time, which helps vaccination centers plan staffing more efficiently and use resources more effectively. Even when additional demand related to influenza outbreaks is taken into account, routine immunization services remain stable, suggesting that adequate capacity can be maintained for emergency situations.

**Discussion:**

By making vaccination schedules more balanced and predictable, this approach offers practical decision support for public health vaccination centers. The findings suggest that more structured scheduling can improve operational efficiency, strengthen preparedness for epidemic outbreaks, and help ensure equitable access to pediatric immunization services.

## 1 Introduction

Recurrent public health emergencies have intensified scrutiny of the scientific precision and rigor of public health administration against the backdrop of infectious disorders sweeping the world (1). Among numerous preventive approaches, vaccination has proven to be one of the most efficient weapons for disease control (2). It is predicted that vaccinations against ten main infections saved around 37 million lives worldwide between 2000 and 2019 (3). Pediatric immunization is particularly crucial since many vaccine-preventable diseases in children can escalate to severe or life-threatening results if left unprotected (4). Meanwhile, due to varying dose requirements, minimum inter-dose intervals, age-dependent administration windows, and co-administration restrictions across vaccines, the growing portfolio of pediatric vaccines and regular updates to immunization recommendations have made scheduling more difficult. The difficulty is most obvious in big and densely populated nations such as China, where meticulous planning and resource allocation are essential to assure timely and fair vaccination delivery. Therefore, in order to maintain high regular coverage while adjusting to changing immunization programs and demand variations, more systematic, optimization-based scheduling support is required.

Currently, childhood vaccination in China is predominantly given through the National Immunization Programme, which focuses on newborns and preschool children (about 0–6/7 years) and extends to booster or catch-up doses for those under 14 years (5). The programme covers important vaccines such as diphtheria-tetanus-pertussis and poliomyelitis, although a growing number of non-programme vaccines with varied schedules and age-specific limitations are increasingly employed in practice. Given Chinas massive population, the public health sector faces continuous obstacles in delivering large-scale immunization programs. In particular, the diversity of vaccines characterized by different dose numbers, inter-dose intervals (6, 7), and permissible age windows substantially increases scheduling complexity, and local Centers for Disease Control and Prevention (CDC) often struggle to allocate appointments and resources efficiently. During epidemic outbreaks, for example, demand for influenza vaccination can increase rapidly beyond the usual service capacity of vaccination clinics, creating a tension between sustaining routine coverage and providing emergency demands. However, present appointment methods usually rely on fixed-period planning and manual changes, which are unsuitable for handling heterogeneous restrictions and rapid demand shocks. Therefore, under different vaccination limits across pediatric age groups, improving vaccination schedules to support both routine immunization and flexible emergency response constitutes an essential capability requirement for public health decision-makers and policymakers.

Research has begun to address this issue. In recent years, various studies have established scheduling or optimization models for pediatric vaccination centers. For instance, Yu et al. (8) constructed an integer programming model to generate personalized vaccination or catch-up schedules for children, accounting for multiple variables including vaccines, age, and historical vaccination records. This approach enhances vaccination efficiency while reducing missed appointments. Similarly, Batoon and Piad (9) developed a vaccine appointment/scheduling system incorporating a “decision support mechanism + geo-tagging” feature. This system automatically recommends required vaccines based on children's vaccination records and generates vaccination appointment schedules. In addition, Guo et al. (10) employed queueing theory to construct a model of the outpatient vaccination service system, analyzing the optimal configuration of service windows (service counters) for different service steps (pre-screening, registration, vaccination) to optimize the overall vaccination process time. When taken as a whole, these studies show how optimization and analytics can improve pediatric vaccination operations. However, most of them either focus on individualized scheduling logic or clinic process design. Less attention has been given to system-level workload balancing and capacity management across a rolling planning horizon, particularly under fluctuating demand conditions.

Another field of research focuses on the overall optimization of vaccine supply chains and distribution networks, particularly during large-scale immunization campaigns or epidemic emergency periods. For instance, Khalilpoor et al. (11) developed an integrated optimization and simulation model for designing COVID-19 vaccine supply chain networks (VSCNs), identifying optimal locations for ultra-low temperature storage centers to ensure efficient vaccine distribution and supply chain resilience. Guo et al. (12) studied how to combine vaccine demand allocation, delivery routes, distribution centers/vaccination locations, and population distribution under large-scale vaccination campaigns/epidemic emergency scenarios. This method tries to decrease travel, distribution, and service costs while maximizing immunization coverage. Recent research have also developed randomized/mixed-integer programming methods for optimizing the construction and distribution networks of pediatric vaccine cold chains, seeking to reduce vaccine wastage, boost delivery efficiency, and ensure vaccine accessibility (13). Furthermore, considering how to prioritize vaccination and optimize age group tactics under conditions of low vaccine supply or scarce resources, Wu et al. (14) developed a multi-objective optimization/age-stratified allocation framework based on epidemiological dynamics and optimization models. This paradigm optimizes resource allocation strategies under fluctuating vaccination efficacy, production capacity, or supply restrictions. Although these contributions significantly advance vaccine logistics and macro-level resource allocation, they mainly operate at the network or population-allocation level. Comparatively fewer studies examine appointment scheduling and workload stabilization within vaccination centers under explicit service-capacity constraints.

Importantly, recent studies increasingly integrate predictive analytics and optimization to improve outpatient scheduling performance. For example, In order to improve waiting times and resource usage, Babayoff et al. (15) created machine-learning-based models for predicting no-shows and service durations. They then integrated these models into appointment scheduling systems. Similarly, Topuz et al. (16) explicitly considered service-capacity restrictions and utilization trade-offs while proposing a data-driven decision-support framework for appointment scheduling and overbooking under unknown attendance behavior. In addition to single-visit scheduling, Han et al. (17) investigated multi-appointment patient scheduling issues where patients need many visits while facing varying resource limitations. Their methodology demonstrates how recurrent appointment scheduling can be done with optimization models while still being operationally feasible. These studies provide important methodological insights for capacity-constrained healthcare scheduling systems. In particular, they demonstrate how predictive modeling and optimization can be combined to enhance resource utilization, stabilize service operations, and manage repeated patient visits over time.

Despite these gains, present research also reveals several limitations. Most models either focus only on vaccine supply chains/distribution systems or are constrained to static pediatric immunization arrangements based on individual patients. Alternatively, they address only appointment scheduling issues under constraints of routine outpatient service capacity. Few systematically integrate routine pediatric immunization with epidemic emergency vaccination into a cohesive paradigm, balancing both steady-state operating capacity and emergency response capability. Therefore, this work aims to propose and verify a complete scheduling optimization model that balances routine childhood immunization with emergency vaccine response. The model allows for complicated real-world factors such dose time windows, vaccination intervals, and facility capacity, whilst automatically altering scheduling techniques during abrupt epidemics. This assures efficient vaccination allocation, minimizes resource wastage, and enhances overall public health coverage quality. Specifically, utilizing a Shenzhen CDC in China as a case study, this research blends routine pediatric vaccinations with predetermined emergency vaccine demands (e.g., influenza) into a unified optimization model. Combined with an ARIMA forecasting model, a CPLEX optimization algorithm creates optimal vaccine schedule plans. This guarantees balanced use of medical resources across immunization locations while conserving sufficient service capacity to respond to possible public health emergencies.

The innovations of this paper lie in:

1. Development of a unified scheduling framework that simultaneously supports routine vaccination operations and epidemic emergency response, boosting the operational resilience of vaccination sites;
2. Incorporation various realistic restrictions identified in real-world vaccination services, boosting the model's viability and applicability;
3. Based on real CDC data, that balanced scheduling can greatly increase resource utilization and service stability.

This paper is constructed as follows: Section 2 covers the technique, describing the construction of the vaccination schedule model and its basic parameters. Section 3 summarizes the results from the case study, comparing the optimal scheduling outcomes with traditional, non-optimized scheduling procedures. Finally, Section 4 analyzes the significance of the results for vaccine management and healthcare resource allocation, proposing recommendations for enhancing vaccination practices in both routine and emergency scenarios.

## 2 Materials and methods

This study develops a mathematical model to conduct numerical experiments and analyses on specific cases, thereby establishing a balanced scheduling scheme generally applicable to disease prevention and control centers and vaccination centers. The model includes three key functions: (1) Determine the earliest and latest dates for administering a specific dose of a particular vaccine to the child based on their age; (2) The specific vaccination date for the child shall be determined based on the time window for the administration of a particular dose of the vaccine; (3) To ensure a fundamentally balanced number of children vaccinated daily within the vaccination scheduling cycle, thereby reserving balanced capacity for vaccination centers each day and securing sufficient vaccination space for contingencies.

In addition to detailing the model creation technique, we further tested the practical application of this model through a case study based on two scenarios from the current childhood immunization campaign at a CDC in Shenzhen, China. These scenarios involve both conventional immunization schedules and concerns for reacting to influenza epidemics.

### 2.1 Model parameters and notation

To further demonstrate the topic and build the model, plausible assumptions have been made regarding important scenarios based on actual study findings, as follows:

1. The target population for immunization covered in this research comprises newborns and young children (aged 0–14 years). Other groups requiring immunization will be recognized as those necessitating vaccination in response to emergency situations.
2. Migratory populations (e.g., migrant children or children who relocate outside the study area) are not considered in this study.
3. This study focuses primarily on Category I vaccines included in the National Immunization Program (e.g., hepatitis B, BCG, polio, DTP, and DT). Category II vaccines-administered voluntarily and at personal expense (e.g., hepatitis A, Hib, influenza, rabies, and COVID-19), as well as alternative vaccines, are assumed to follow scheduling rules comparable to those of Category I vaccines.
4. Missed appointments after scheduling are examples of non-compliance issues that are not specifically taken into account.
5. The vaccination center operates six days per week (Monday to Saturday) and is closed on Sundays. All scheduled vaccination days are assumed to be regular operating days.

The notation used in the proposed model is summarized in Table 1.

**Table 1:** Model parameters and notations.

**Notation**	**Description**
**Parameters**	
*N*	Set of children
*M*	Set of vaccine types
*T*	Set of scheduling days within a scheduling cycle
*F*	Set of all children's ages
*K*	Set of vaccine doses
*D*	Daily service capacity of the vaccination center
*m*_*t*_	Epidemic vaccination demand on day *t*
*w*_*ijk*_	The *i*-th child requires the *k*-th dose of the *j*-th vaccine (1 if required, 0 otherwise).
*f*_*i*_	The age of the *i*-th child
*l*_*jk*_	The minimum age for the *k*-th dose of the *j*-th vaccine
*s*_*jk*_	The minimum interval between the *k*-th dose and the previous dose of the j-th vaccine
*p*_*ijk*_	The earliest administration time for the *k*-th dose of the *j*-th vaccine for the *i*-th child, where $p_{ijk}=\{\begin{array}{r} l_{jk}\times w_{ijk}-f_{i},f_{i}+s_{jk}\times w_{ijk}<l_{jk}\\ s_{jk},f_{i}+s_{jk}\times w_{ijk}\geq l_{jk} \end{array}$
*d*_*ijk*_	The scheduling delay for each child-dose
$\bar{v}$	average daily vaccination volume
*L*	scheduling cycle
*R*	The range of daily volumes after the schedule reaches steady state
*o*_*jk*_	The latest allowable age for administration of the *k*-th dose of the *j*-th vaccine
*q*_*ijk*_	The latest allowable administration time for the *k*-th dose of the *j*-th vaccine for the i-th child
*h*_*t*_	The number of children already scheduled per day at the initial stage of scheduling
*Y*	The balanced daily vaccination workload
**Decision Variables**	
*x*_*ijkt*_	The *k*-th dose of vaccine *j* for child *i* is scheduled on day *t* (a binary variable: 1 if scheduled on that day, 0 otherwise)
*t*_*ijk*_	The specific time of the *k*-th dose of vaccine *j* for child *i* ($t_{ijk}=\underset{t}{\sum }x_{ijkt}\times t$)

This study primarily establishes vaccination scheduling for the following three scenarios: Firstly, to arrange balanced intervals for subsequent doses of a specific vaccine series for children who have received a particular dose that day. Secondly, to schedule the administration of a specific dose of a vaccine for children entering the scheduling cycle on that day. Thirdly, to schedule the administration of a specific dose of a vaccine for newborn infants born each day. The scheduling horizon spans from day 28 to day 60, resulting in a 33-day scheduling cycle. This design reflects the minimum inter-dose interval of 28 days required by most vaccines and allows vaccination appointments to be scheduled approximately one month in advance. Scheduling is conducted using a rolling-horizon approach, whereby vaccination decisions are updated daily based on newly arriving children and previously scheduled appointments. For children whose feasible vaccination time windows initially fall outside the current cycle, scheduling is deferred until their age enters the active scheduling window.

### 2.2 Optimization model

The conventional vaccination optimization model is formulated as a integer programming problem, as shown in Equations 1–6. Objective function (1) indicates that the number of individuals vaccinated each day should be balanced, meaning the total number vaccinated each day cycles. Days with fewer vaccinations will have eligible individuals rescheduled, while days with higher vaccination numbers will temporarily not schedule additional individuals (maximizing the day with the fewest vaccinations; the total number vaccinated includes both previously scheduled individuals and those scheduled during this cycle). This objective ensures that the least-loaded day in each cycle is maximized, thereby smoothing daily workload fluctuations. Constraints (2) stipulates that the child's vaccination time for this vaccine must be no earlier than the earliest possible vaccination time. Constraints (3) specifies that the latest possible vaccination time for this vaccine must be no later than the latest permissible vaccination time (i.e., the vaccination time must fall within the prescribed time window). Constraints (4) denotes the specific vaccination date for the child (e.g., *x*_*ijk*29_ = 1, *t*_*ijk*_ = 1 × 29 = 29, meaning this dose of the vaccine is scheduled for the child on the 29th day). Constraints (5) stipulates that no child may receive more than two vaccine doses within a single day. Constraints (6) specifies that for any given child, the same dose of the same vaccine must be administered on the same day and cannot be split across two days.

The objective function is as follows:


$$\begin{array}{l} Y=\max\left[\underset{t}{\min}\left(\overset{n}{\underset{i=1}{\sum }}\overset{m}{\underset{j=1}{\sum }}\overset{K}{\underset{k=1}{\sum }}x_{ijkt}\times w_{ijk}+h_{t}\right)\right] \end{array}$$ (1)

The constraints are as follows:


$$\begin{array}{l} 0\leq p_{ijk}\leq t_{ijk}\;\forall i\in N,j\in M,k\in K,2 \end{array}$$ (2)

$$\begin{array}{l} t_{ijk}\leq q_{ijk}\;\forall i\in N,j\in M,k\in K,3 \end{array}$$ (3)

$$\begin{array}{l} t_{ijk}=\underset{t}{\sum }x_{ijkt}\times t\;\forall i\in N,j\in M,k\in K,t\in T,4 \end{array}$$ (4)

$$\begin{array}{l} \overset{m}{\underset{j=1}{\sum }}\overset{K}{\underset{k=1}{\sum }}x_{ijkt}\leq 2\;\forall i\in N,j\in M,k\in K,t\in T,5 \end{array}$$ (5)

$$\begin{array}{l} \overset{T}{\underset{t=1}{\sum }}x_{ijkt}=1\;\forall i\in N,j\in M,k\in K,t\in T.6 \end{array}$$ (6)

**Figure 1:**
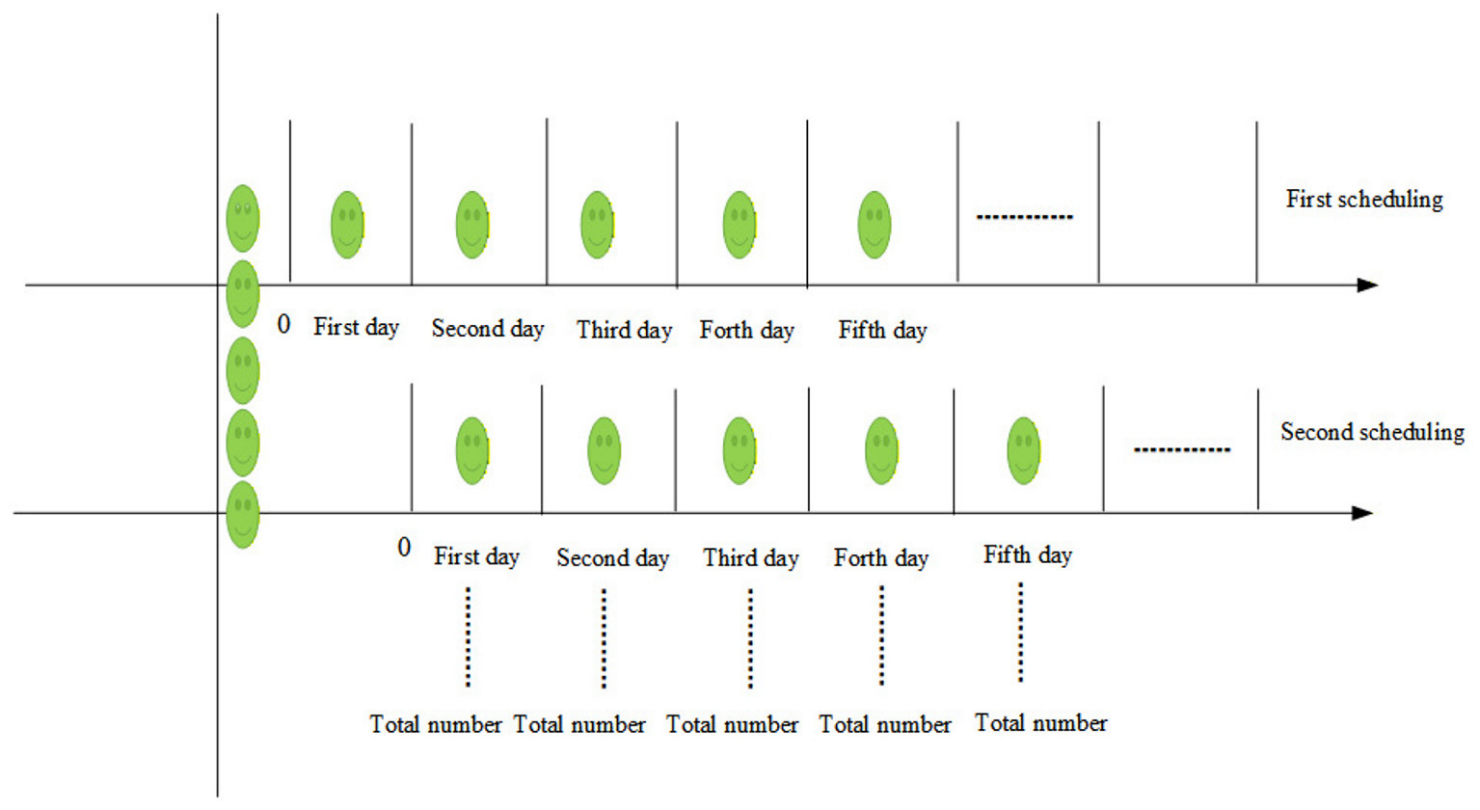
Scheduling cycle recursive diagram.

Among these, *x*_*ijkt*_∈{0, 1}, *t*_*ijk*_>1; *i*∈{1, 2, ⋯ , *n*}, *j*∈{1, 2, ⋯ , *m*}, *k*∈{1, 2, 3, 4}, *t*∈{1, 2, ⋯ , *T*}. In addition, to evaluate the timeliness of the resulting schedule, we define the scheduling delay for each child-dose combination as follows:


$$\begin{array}{l} d_{ijk}=t_{ijk}-p_{ijk} \end{array}$$

where *t*_*ijk*_ is the optimized appointment date assigned by the model and *p*_*ijk*_ is the earliest permissible vaccination date determined by age eligibility and inter-dose interval requirements. A delay of zero indicates that the child is scheduled at the earliest possible date, whereas a positive delay reflects deferral within the admissible time window [*p*_*ijk*_, *q*_*ijk*_]. This metric enables assessment of whether the workload-balancing objective inadvertently compromises vaccination timeliness. The scheduling cycle recursiveness is illustrated in Figure 1.

According to the scheduling recursive rule, the final number of vaccination doses scheduled for a given day is the total number of children ultimately scheduled for vaccination on that day after the recursive cycle concludes (i.e., after one cycle period has elapsed for that day).

Furthermore, when special epidemics occur, such as seasonal influenza, it is necessary to reserve corresponding vaccination service capacity based on the spread and control of the epidemic. The objective function is then:


$$\begin{array}{l} Y=\max\left[\underset{t}{\min}\left(\overset{n}{\underset{i=1}{\sum }}\overset{m}{\underset{j=1}{\sum }}\overset{K}{\underset{k=1}{\sum }}x_{ijkt}\times w_{ijk}+h_{t}+m_{t}\right)\right]7 \end{array}$$ (7)

Moreover, the constraints are extended to include:


$$\begin{array}{l} \overset{n}{\underset{i=1}{\sum }}\overset{m}{\underset{j=1}{\sum }}\overset{K}{\underset{k=1}{\sum }}x_{ijkt}+h_{t}+m_{t}\leq D\;\forall t\in T8 \end{array}$$ (8)

Where *D* represents the daily vaccination service capacity of the vaccination center (i.e., the maximum number of individuals who can be vaccinated simultaneously), and *m*_*t*_ denotes the projected daily number of individuals requiring vaccination following the sudden emergence of a specific epidemic. Objective function (7) indicates that during an epidemic requiring vaccination, routine Category I vaccines and vaccines required for the special epidemic should be scheduled in a balanced manner (i.e., sufficient vaccination service capacity should be reserved for emergency vaccination scenarios). Constraints (8) indicates that the daily number of vaccinations scheduled must not exceed the vaccination service capacity of the vaccination professionals.

## 3 Results

This study selected a vaccination center in Nanshan District, Shenzhen, China as a case study for scheduling vaccinations among pediatric attendees. Through field research, we collected daily vaccination records for children aged 0-14 years from October to December 2017, together with influenza vaccination data from June to December 2017 to capture seasonal and outbreak-related demand surges. The dataset includes each vaccine type and administered dose number. Specifically, all children's vaccination information is derived from the CDC's appointment allocation results based on a first-come, first-served rule. A typical operating day was chosen as the initial point to initialize the rolling-horizon scheduling process, based on which a queue was organized for children attending on that day; subsequent cohorts were accommodated via daily rolling updates. Analysis of the scheduling results has yielded a feasible and operationally sound vaccination programme. In addition, vaccination constraints were derived from the 2016 Edition of the Immunization Work Specifications (18) and encoded as age-restricted time windows and inter-dose interval requirements for Category I vaccines. In general, each dose must fall within a permissible age window and satisfy a minimum interval from the previous dose. For example, the first dose of hepatitis B vaccine (HepB) should be administered within 24 hours after birth, and subsequent doses require intervals longer than 28 days; BCG vaccination must be completed before 3 months of age.

In the case analysis, This study propose examine the outcomes of vaccination schedules under two scenarios: one where no influenza outbreak occurs, and another where an influenza outbreak does occur.

### 3.1 Vaccination scheduling results under non-epidemic conditions

In the case study analysis and solution process, the types of existing vaccines and their corresponding administration requirements were inputted. The scheduling cycle runs from day 28 to day 60, with one scheduling session occurring per cycle. Considering the interval between two doses and the need to schedule vaccines included in the cycle one month in advance, scheduling is balanced after 28 days and conducted on a rolling basis (assuming an initial scheduling state where *h*_*t*_ = 0; each subsequent scheduling session balances the daily vaccination numbers based on the previous session, meaning rolling scheduling commences from the second session onwards, where *h*_*t*_≠0). Each rolling schedule advances one day forward (e.g., the day following the first schedule becomes the first day of the second schedule, and so forth). If a vaccines guideline-based feasible time window extends beyond the current cycle, its admissible dates are truncated to days 30-60 to comply with the one-month advance-scheduling principle. The resulting integer optimization problem was solved using CPLEX to determine (i) the appointment date for the next dose of a given vaccine among children arriving daily, and (ii) the appointment dates for specific doses of children already included in the current cycle; identical procedures were applied to other vaccine types (e.g., triple and quadruple vaccines). For the detailed scheduling plan, please refer to Appendix A (with 45 scheduled instances), which summarizes scheduling outcomes at five-day intervals, while the consolidated schedule under non-epidemic conditions is presented in Table 2. Specifically, detailed CPLEX solution code is provided in Appendix B.

**Table 2:** Scheduling results excluding pandemic considerations.

**Day**	28	29	30	31	32	33	34	35	36	37	38	39	40	41
**Daily vaccination volume**	2	2	2	4	5	4	5	6	6	7	7	8	9	10
**Day**	42	43	44	45	46	47	48	49	50	51	52	53	54	55
**Daily vaccination volume**	10	11	12	12	12	13	13	13	13	14	14	14	15	16
**Day**	56	57	58	59	60	61	62	63	64	65	66	67	68	69
**Daily vaccination volume**	15	16	16	17	18	17	17	18	17	18	17	18	17	20
**Day**	70	71	72	73	74	75	76	77	78	79	80	81	82	83
**Daily vaccination volume**	18	18	17	19	19	17	18	19	19	19	19	18	18	19

Table 2 presents the final scheduling results under non-epidemic conditions, specifically the daily vaccination capacity of each vaccination site. Between days 28 and 60, the daily allocation of individuals is low because the schedule did not complete a full cycle during this period (due to the influence of the initial state). Once the schedule reached day 60, the daily data gradually began to satisfy a full cycle. Consequently, from day 60 onwards, the daily scheduling results progressively stabilized into static data, ceasing to roll through the cycle. Table 2 demonstrates that beyond day 60, the daily scheduling approaches equilibrium, with the total fluctuating minimally between 17 and 20.

**Figure 2:**
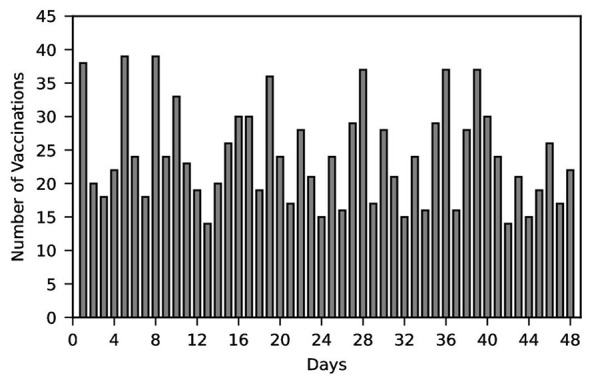
The number of people coming for vaccination each day.

This section further conducts a functional analysis and effectiveness assessment of the vaccination scheduling outcomes. Firstly, based on daily vaccination figures from October to December 2017 at a Shenzhen vaccination center, the daily routine vaccination numbers during periods without influenza outbreaks were determined, as shown in Figure 2.

**Figure 3:**
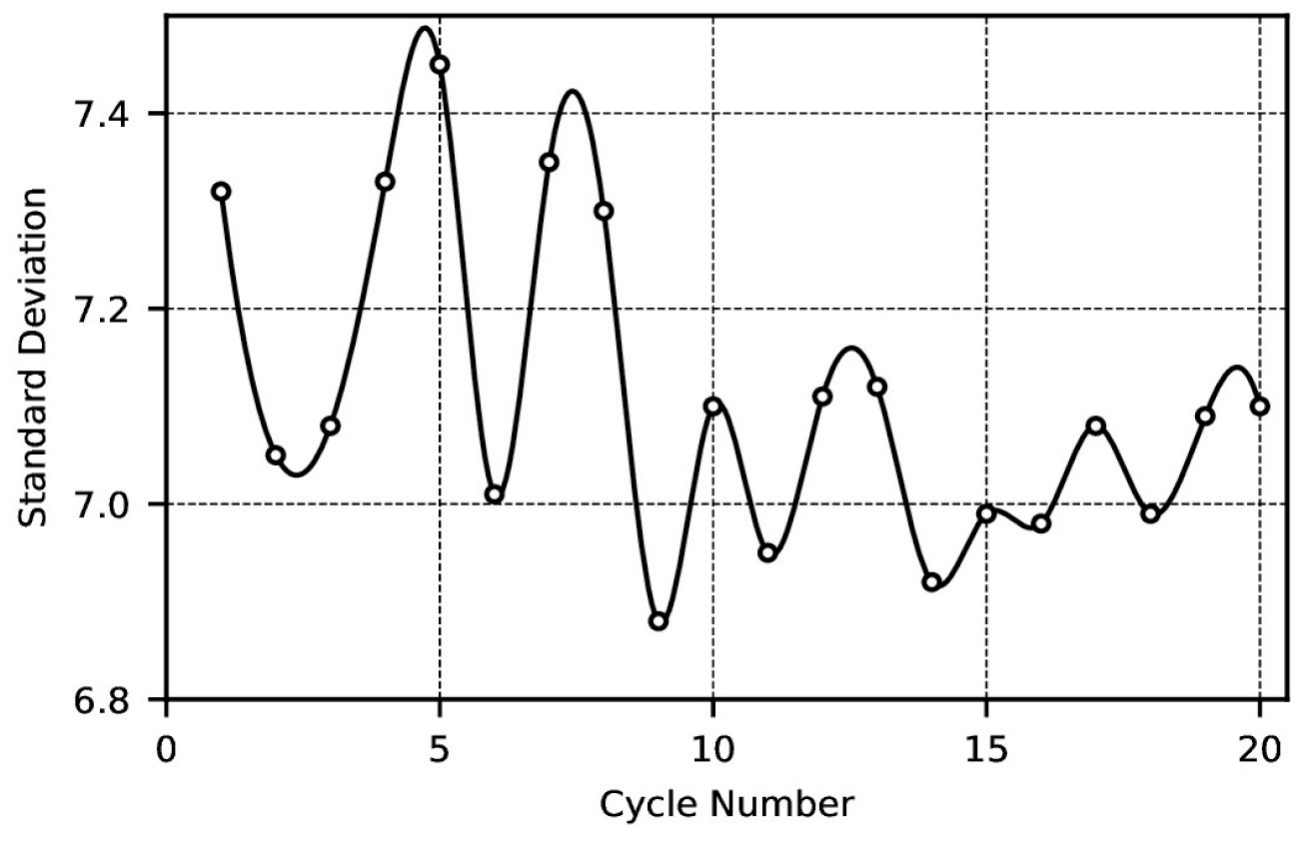
Standard deviation of the number of people attending vaccination appointments every 33 days.

**Figure 4:**
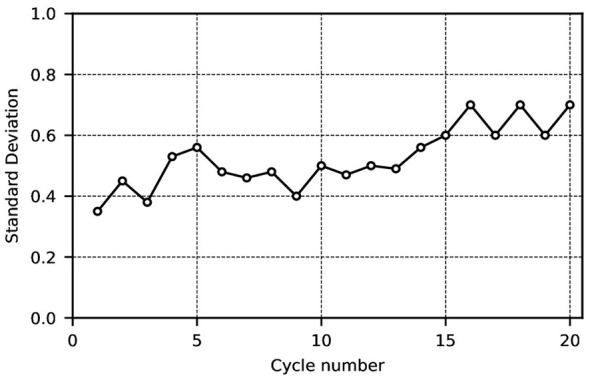
Standard deviation after each balanced scheduling.

As shown in Figure 2, due to the absence of corresponding scheduling rules at vaccination centers, individuals largely attend centers at random times for vaccination. This results in significant daily variations in the daily vaccination numbers at each center. Subsequently, a comparison of the standard deviation in vaccination numbers before and after implementing the scheduling system yielded Figures 3, 4.

Figure 3 depicts the standard deviation of the number of individuals vaccinated per 33-day period (as the scheduling cycle range spans 28-60 days, each cycle comprises 33 days). As evident from Figure 3, the corresponding standard deviation for each 33-day period is substantial, ranging between 6 and 7.5. This disparity hinders the balanced utilization of vaccination center resources. On days with high vaccination demand, service capacity is fully occupied, leaving no capacity to address unforeseen circumstances or provide vaccinations. Conversely, on days with low demand, numerous vaccination professionals remain idle, resulting in resource wastage, diminished service quality, and reduced satisfaction levels.

Figure 4 illustrates the standard deviation for each cycle following every scheduling run. As shown in Figure 4, the standard deviation for each cycle after scheduling is relatively small, fluctuating primarily between 0 and 1. This indicates that the daily number of vaccinations scheduled is fairly balanced following each scheduling run. When the number of individuals attending for vaccination on a given day is sufficient to achieve an even distribution across the subsequent cycle, the standard deviation approaches zero. Should the number attending the following day prove insufficient to balance the upcoming cycle, the standard deviation will exceed the previous value. It will decrease again when the subsequent scheduling cycle can restore an even distribution, though the standard deviation consistently fluctuates within a narrow range.

**Figure 5:**
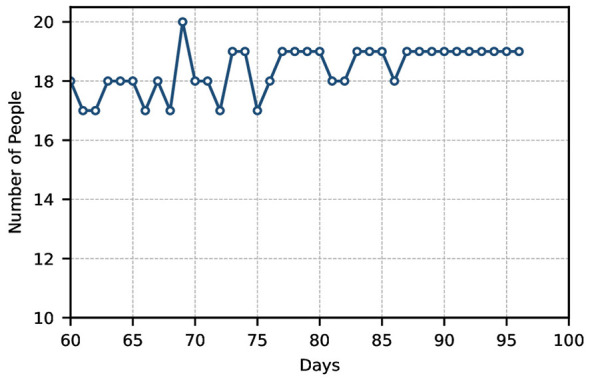
Scheduling results after one cycle.

**Figure 6:**
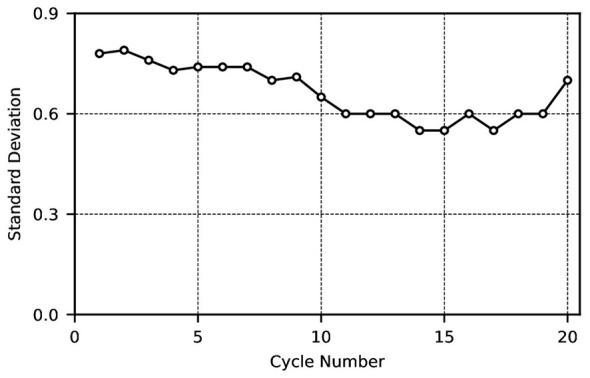
Standard deviation for each cycle following completion of the schedule.

Further analysis of the scheduling results yields the outcomes after one cycle and the standard deviation for each cycle following completion of the schedule, as shown in Figures 5, 6.

Figure 5 shows the number of people scheduled for vaccination after each daily cycle. As seen in Figure 5, after one cycle, the daily vaccination schedule tends to stabilize at roughly equal numbers. The chart indicates that the final daily allocation converges between 17 and 20, achieving equilibrium. Figure 6 illustrates the standard deviation for each cycle following completion of the scheduling process. It is evident from the graph that the standard deviation is relatively small (fluctuating primarily between 0 and 1).

#### 3.1.1 Analysis of scheduling timeliness

Although the aforementioned analysis indicates that the proposed model can effectively stabilize the daily workload of vaccine inoculation, it remains to be determined whether this balance is achieved at the expense of inoculation timeliness. To this end, we further analyze the timeliness of vaccination scheduling based on the scheduling results.

By construction, the delay *d*_*ijk*_ is non-negative and bounded above by the scheduling cycle length. Since the active scheduling window spans days 28 to 60, for any child–dose pair whose earliest eligible date *p*_*ijk*_ falls within the current cycle, the maximum possible delay is at most 32 days. In practice, however, two structural features of the model ensure that actual delays are substantially smaller than this theoretical bound.

First, the max–min balancing objective (1) drives the optimizer to assign vaccinations preferentially to days with lower workloads. Consider a child whose earliest eligible date *p*_*ijk*_ coincides with a day that currently carries a below-average load. In this case, assigning the child to day *p*_*ijk*_ directly contributes to raising the minimum daily count, which is exactly what the objective function seeks to maximize. Consequently, such children are scheduled with zero delay. Conversely, when *p*_*ijk*_ falls on a day that is already at or above the average load, the optimizer shifts the appointment to the nearest under-loaded day. Given the narrow range of daily workloads observed after convergence (17–20, as shown in Table 2), such shifts are necessarily small in magnitude.

Second, the rolling-horizon design ensures that children whose admissible time windows extend beyond the current scheduling cycle are automatically reconsidered in subsequent cycles, preventing any indefinite deferral of vaccination appointments.

Therefore, We $\bar{v}$ denote the average daily vaccination volume and *R* = *v*_max_−*v*_min_ denote the range of daily volumes after the schedule reaches steady state. For the earliest eligible child, when the vaccination date *p*_*ijk*_ falls within the current vaccination cycle *L*, the scheduling delay satisfies:


$$\begin{array}{l} d_{ijk}\leq \frac{R\cdot L}{\bar{v}} \end{array}$$

From Table 2, the steady-state daily vaccination volume fluctuates between *v*_min_ = 17 and *v*_max_ = 20, yielding a range of *R* = 3 and an approximate mean of $\bar{v}\approx 18.4$. The cycle length is *L* = 33 days. We have:


$$\begin{array}{l} d_{ijk}\leq \frac{3\times 33}{18.4}=5.38=6\text{days} \end{array}$$

This indicates that even in the worst case, the scheduling delay does not exceed approximately 6 days under non-epidemic conditions. In addition, In the detailed scheduling tables (Tables 6–10), each scheduling run distributes newly arriving children across the 33-day cycle. In steady-state runs (e.g., Runs 41–45 in Table 10), the overwhelming majority of daily “Result” entries are 0 or 1, indicating that each new scheduling run adds at most one additional vaccination per day. This highly uniform distribution pattern implies that the optimizer consistently finds available capacity close to each child's earliest eligible date. Furthermore, the standard deviation of daily vaccination volumes following each scheduling run remains between 0 and 1 (as shown in Figures 4, 6). Since any systematic deferral of children from early to later days in the cycle would inflate the standard deviation of daily totals, the persistently low standard deviation provides indirect but robust evidence that the mean scheduling delay is small.

Taken together, these findings indicate that the max–min workload-balancing objective does not introduce substantial timeliness penalties. The vast majority of children are scheduled within a few days of their earliest eligible date, and the inherent structure of the optimization model provides natural safeguards against excessive delay.

### 3.2 Vaccination scheduling results under epidemic conditions

**Figure 7:**
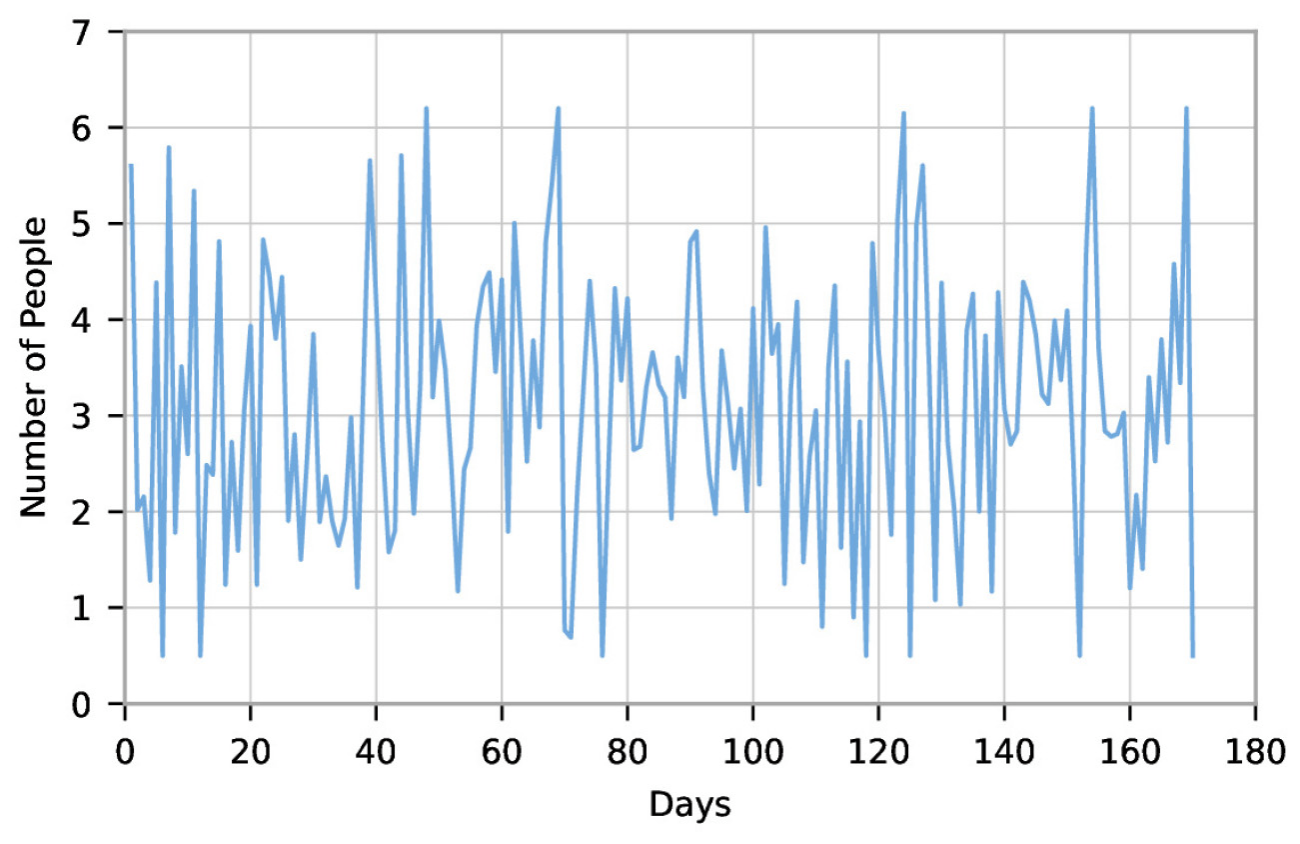
Daily vaccination numbers against influenza.

When an epidemic occurs (such as an influenza outbreak), the primary step is to forecast the number of individuals requiring vaccination, thereby determining the contingency capacity for vaccination services that should be reserved. To estimate epidemic-related vaccination demand, influenza vaccination records from June to December 2017 were analyzed. As shown in Figure 7, the observed time series exhibits both temporal continuity and random fluctuations, making it suitable for time series modeling. This study further employs the ARIMA(p,d,q) model to forecast vaccination demand. All data processing, model estimation, and forecasting tasks were completed using EVIEWS 8.0 software.

The ARIMA model forecasts future values based on multiple historical data points. Let *y*_*t*_ denote the number of epidemic cases in period *t*; its fundamental expression is:


$$\begin{array}{l} y_{t}=\varphi_{1}y_{t-1}+\varphi_{2}y_{t-2}+\cdots +\varphi_{p}y_{t-p}+\varepsilon_{t}-\theta_{1}\varepsilon_{t-1}-\cdots -\theta_{q}\varepsilon_{t-q}9 \end{array}$$ (9)

In Equation 9, *p* denotes the autoregressive order, *q* represents the moving average order, *d* indicates the differencing order, φ_1_, …φ_*p*_ are non-zero parameters, and *y*_*t*_ is a stationary sequence with zero mean.

The augmented DickeyCFuller (ADF) test on the original series gives DF = -11.44376, which is below the 1%, 5%, and 10% critical values (-3.469, -2.8786, and -2.575954). Therefore, the null hypothesis of a unit root is rejected, and the series is stationary. In addition, the autocorrelation coefficients fluctuate predominantly around zero, suggesting weak persistence and supporting a low-order ARIMA specification. The parameter estimation and diagnostic results of the fitted ARIMA(1,0,1) model are presented in Table 3.

**Table 3:** Estimation results of the ARIMA(1,0,1) model.

**Variable**	**Coefficient**	**Std.Error**	**t-Statistic**	**Prob**.
C	3.177536	0.297727	10.67264	0.0000
AR(1)	0.956859	0.034421	27.79846	0.0000
MA(1)	−0.907706	0.055969	−16.21805	0.0000
R-squared	0.074101	Mean dependent var		3.029762
Adjusted R-squared	0.062877	S.D. dependent var		1.668394
S.E. of regression	1.615091	Akaike info criterion		3.814355
Sum squared resid	430.4055	Schwarz criterion		3.870140
Log likelihood	−317.4058	Hannan-Quinn criter.		3.836995
F-statistic	6.602544	Durbin-Watson stat		2.000578
Prob(F-statistic)	0.001744			
Inverted AR Roots	.96			
Inverted MA Roots	.91			

From Table 3, it can be seen that both the AR(1) and MA(1) coefficients are statistically significant (*p* < 0.001), indicating a strong low-order dependence structure. The inverted AR and MA roots are smaller than 1, confirming that the fitted model is stationary and invertible. Furthermore, the formula for determining the required number of influenza vaccine doses for the forthcoming cycle can be derived as follows:


$$\begin{array}{l} y_{t}=0.957y_{t-1}+\varepsilon_{t}-0.908\varepsilon_{t-1}+3.1810 \end{array}$$ (10)

In Equation (10), *t* denotes a specific day in the time series, *y*_*t*_ represents the number of individuals requiring vaccination on day *t*, and ε_*t*_ indicates the moving average of *y*_*t*_.

Based on the forecast results, a balanced scheduling plan for Category I vaccinations and influenza vaccinations was formulated, with the scheduling outcomes presented in Table 4.

**Table 4:** Schedule optimization results under epidemic scenarios.

**Day**	28	29	30	31	32	33	34	35	36	37	38	39	40	41
**Daily schedule for category 1 vaccines**	1	1	3	3	4	4	4	5	6	8	9	8	8	8
**Influenza vaccine vaccination volume**	2	3	2	3	2	3	2	3	2	3	2	3	3	3
**Total vaccination volume**	3	4	5	6	6	7	6	8	8	11	11	11	11	11
**Day**	42	43	44	45	46	47	48	49	50	51	52	53	54	55
**Daily schedule for category 1 vaccines**	9	9	11	11	11	11	10	11	13	13	14	14	15	16
**Influenza vaccine vaccination volume**	2	5	3	3	3	3	5	5	3	4	3	4	3	2
**Total vaccination volume**	11	14	14	14	14	14	15	16	16	17	17	18	18	18
**Day**	56	57	58	59	60	61	62	63	64	65	66	67	68	69
**Daily schedule for category 1 vaccines**	15	17	16	17	18	16	15	18	18	19	19	17	18	17
**Influenza vaccine vaccination volume**	3	3	3	4	3	4	4	2	3	2	3	4	4	3
**Total vaccination volume**	18	20	19	21	21	20	19	20	21	21	21	21	22	20
**Day**	70	71	72	73	74	75	76	77	78	79	80	81	82	83
**Daily schedule for category 1 vaccines**	17	17	19	19	19	19	17	18	19	19	18	19	18	19
**Influenza vaccine vaccination volume**	5	3	3	2	3	4	3	4	3	4	3	3	4	3
**Total vaccination volume**	22	20	22	21	22	23	20	22	22	23	21	22	22	22

As Table 4 demonstrates, even when accounting for epidemic outbreaks, the daily scheduling totals remain largely balanced, indicating the model's robust stability. According to Table 4, after 60 days, daily scheduling approaches equilibrium, with the number of daily vaccinations fluctuating between 20 and 22. Given this balanced daily distribution of influenza vaccinations, the scheduling outcomes for Category I vaccines are minimally affected. These scheduling outcomes enable disease control centers and vaccination sites to allocate professional vaccinators' shifts according to daily vaccination volumes, thereby avoiding resource wastage.

Currently, each professional at Shenzhen Nanshan Disease Control Center can administer vaccinations to approximately 80 individuals daily. Consequently, balanced daily vaccination capacity can be reserved for unexpected demand. Based on daily vaccination numbers, vaccination centers need only assign one professional vaccinator per day.

In addition, we extend the delay analysis of Section 3.3.1 to the epidemic scenario. From Table 4, the steady-state daily total vaccination volume (routine plus influenza) fluctuates between 20 and 23 after day 60, with Category I vaccine volumes remaining within the range of 17–19. The range of total daily volumes is *R* = 3 and the mean is approximately $\bar{v}\approx 21.3$, yielding a worst-case delay bound of (3 × 33)/21.3 = 4.65 ≈ 5 days. This is slightly tighter than the non-epidemic bound (6 days), because the higher total daily volume reduces the relative magnitude of day-to-day variation.

More importantly, the Category I vaccination schedules in Table 4 remain close to those in Table 2 (steady-state range 17–19 vs. 17–20), suggesting that the introduction of influenza vaccination demand does not materially degrade the timeliness of routine childhood immunization.

#### 3.2.1 Sensitivity analysis of epidemic demand forecasts

Since the ARIMA-based forecast of epidemic demand *m*_*t*_ is inherently a point estimate, it is important to assess the sensitivity of the scheduling outcomes to forecast uncertainty. To this end, we conduct a scenario-based sensitivity analysis by perturbing the baseline forecast using a scaling factor δ, δ∈{0.2, 0.5}. Specifically, three demand scenarios are considered:

- A *low-demand scenario* in which the daily epidemic demand is reduced to *m*_*t*_·(1−δ), representing an overestimation of the outbreak severity;
- The *baseline scenario* corresponding to the original ARIMA forecast *m*_*t*_;
- A *high-demand scenario* in which the daily demand is inflated to *m*_*t*_·(1+δ), reflecting an underestimation of epidemic intensity.

Table 5 summarizes the key scheduling performance indicators under each scenario after the schedule reaches steady state (from day 60 onward).

**Table 5:** Sensitivity analysis of scheduling outcomes under varying epidemic demand scenarios.

**Scenario**	**δ**	**Avg. daily influenza demand**	**Cat. I vaccinerange**	**Total daily volume range**	**Std. dev. per cycle**
Low	0.5	≈1.5	17–20	18–21	0.3–0.8
Low	0.2	≈2.4	17–19	19–22	0.3–0.9
**Baseline**	**0**	**≈3.0**	**17–19**	**20–23**	**0.4–0.9**
High	0.2	≈3.6	17–19	21–24	0.4–1.0
High	0.5	≈4.5	16–19	22–25	0.5–1.2

All values are computed from steady-state scheduling results (day 60 onward). Fractional demand values are rounded up to the nearest integer. Bolded values indicate results under the baseline scenario corresponding to the original ARIMA forecast of influenza demand.

The results in Table 5 indicate that: (1) the Category I (routine) vaccination schedule remains remarkably stable across all demand scenarios. Regardless of whether epidemic demand is reduced by 50% or increased by 50%, the steady-state daily volume for routine vaccines consistently falls within the range of 16–20, closely matching the baseline range of 17–19. This stability indicates that the proposed scheduling framework effectively insulates routine immunization from fluctuations in epidemic demand, which is a critical property for maintaining continuous pediatric vaccination services. (2) The total daily vaccination volume adjusts proportionally to the epidemic demand level, as expected, but the day-to-day variability remains well controlled. The per-cycle standard deviation increases only modestly under the high-demand scenario (δ = 0.5), rising from approximately 0.4–0.9 in the baseline to 0.5–1.2, confirming that workload balance is preserved even under substantial demand surges; (3) Under the high-demand scenario with δ = 0.5, the total daily vaccination volume reaches 22–25. Given that each professional vaccinator at the Shenzhen Nanshan CDC can administer vaccinations to approximately 80 individuals per day, the service capacity remains adequate even under the most pessimistic demand assumption. However, if more extreme epidemic scenarios are anticipated (e.g., δ>0.5), additional capacity planning measures—such as supplementary staffing or extended operating hours—may become necessary.

Overall, the sensitivity analysis confirms that the proposed scheduling model is robust to realistic levels of epidemic demand uncertainty, maintaining balanced workloads, adequate routine vaccination coverage, and acceptable timeliness across all tested scenarios.

## 4 Conclusions

Public health systems continue to face significant challenges in ensuring the efficient and fair provision of vaccination services, especially in light of the growing complexity of pediatric immunization regimens and the ongoing threat of epidemics. Vaccination, in contrast to many other healthcare services, necessitates rigorous adherence to age eligibility, dose intervals, and schedule limits, which significantly complicates resource planning and service management.

In order to meet various restrictions during the immunization process, especially the catch-up vaccination of children who have missed their scheduled visits, this work proposes and evaluates an optimized scheduling model for childhood vaccination. A integer programming-based scheduling optimization model was created using a vaccination center in Shenzhen, China as a case study. This model takes into account limitations like age restrictions, the necessary time between immunization doses, and the service capability of vaccination facilities. This methodology optimizes daily vaccination counts, assuring logical resource allocation and effective utilization at vaccination stations, and accurately creates customized catch-up programs for children who have missed their immunization appointments. The optimized plan shows notable improvements in vaccination coverage, resource utilization efficiency, and service quality as compared to conventional random scheduling techniques. According to research, using this optimization model improves vaccination equity and resource use while also lowering the proportion of children who miss doses. This is especially important from a practical standpoint during widespread immunization programs. The model's adaptability and scalability guarantee that it can offer efficient scheduling support even during unexpected outbreaks during epidemic emergency stages. Furthermore, the model's adaptable form enables its implementation in various vaccination contexts, such as adult immunization campaigns or seasonal flu vaccination programs, even though this work focuses on kid vaccination.

From a public health management perspective, the findings of this study provide practical decision-support tools for vaccination centers and public health administrators. First, the proposed optimization model enables vaccination centers to generate vaccination appointment schedules for all children attending on a given day in a single planning process, thereby reducing reliance on manual scheduling. Second, balanced daily vaccination volumes allow managers to determine staffing requirements more accurately and allocate vaccinators duty rosters more efficiently. Third, by aligning vaccination schedules with expected demand, vaccination centers can better plan vaccine procurement and inventory levels, contributing to cost reduction and supply chain efficiency. Although the present study focuses on pediatric vaccination, the proposed framework is readily extendable to other immunization contexts, such as adult vaccination campaigns or seasonal influenza programs.

Furthermore, although having a theoretical foundation derived from a thorough literature review and field research, this study nevertheless has inherent limitations because of the subject matter's inherent complexity and the impact of some subjective aspects. First, the scheduling model is formulated as a integer programming problem. Although computationally efficient under the operational scale considered in this study, integer programming models may become computationally demanding in substantially larger vaccination systems with higher arrival volumes or multi-center coordination requirements. Future research may explore decomposition strategies, heuristic algorithms, or parallel computing techniques to enhance scalability in large-scale public health settings. Second, although the model takes into consideration the majority of routine and emergency vaccination requirements, it ignores additional factors such as vaccine hesitancy, no-show appointments, or appointment cancellations. In addition, the current model does not directly encode age-based priority or timeliness preferences. Future research could incorporate explicit delay penalties or age-group-dependent maximum delay constraints into the objective function, enabling decision-makers to directly control the trade-off between workload balance and vaccination timeliness. For instance, adding a weighted penalty term $\underset{i,j,k}{\sum }\alpha_{jk}\cdot d_{ijk}$ to the objective function would allow prioritization of time-sensitive vaccines (e.g., those with narrow age windows or known efficacy decay) while maintaining overall workload balance. Such extensions would further strengthen alignment with immunization priority principles and constitute an important direction for future research. These further factors could be included in future studies to improve the model and increase its applicability.

Additionally, future studies could explore the integration of stochastic demand models or multi-objective optimization approaches to address uncertainties in vaccination demand during epidemics.

## Data Availability

The raw data supporting the conclusions of this article will be made available by the authors, without undue reservation.
