# Supplementary material for: Decision-making for pediatric vaccination scheduling under multiple constraints: a case study from China✰
Source: Front Public Health. 2026 Mar 6;14:1768367. doi: 10.3389/fpubh.2026.1768367 (PMC13002810; doi:10.3389/fpubh.2026.1768367)
Supplement: Supplementary file 1 [file Table_1.pdf]

Appendix A

In this appendix, we present the results of the routine vaccination scheduling every five days in the absence of an influenza epidemic. The number of scheduling cycles refers to the number of times the cycle is repeated (one cycle is completed when scheduling is performed for the number of individuals attending on that day). Accumulation occurs because scheduling is balanced based on the previous day's allocation. Thus, the cumulative value represents the sum of the number of people scheduled in the previous cycle plus the number scheduled in the current cycle, accumulating sequentially. The accumulation concludes (after one cycle per day) to yield the final total number of vaccinations scheduled for that day. As the initial scheduling lacks a preceding cycle, no accumulation occurs. From the second balanced scheduling onwards, accumulation begins based on the previous allocation.

Appendix B

The pseudocode for the CPLEX solution process is as follows:

```
maximize Y;
subject to {
    forall (l in day) {
        Y <= sum(i in children, j in vaccine) x[i][j][l]*w[i][j] + h[l];
    }
subject to {
    forall (i in children, j in vaccine, k in number)
        if (f[i] + s[j][k]*w[i][j][k] < l[j][k])
            p[i][j][k] == l[j][k]*w[i][j][k] - f[i];
            p[i][j][k] == s[j][k];
        forall (i in children, j in vaccine, k in number)
        q[i][j][k] == o[j][k]*w[i][j][k] - f[i];
        }
        forall (i in children, j in vaccine)
        {
        p[i][j] <= t[i][j];
        t[i][j] <= q[i][j];
        }
        forall (i in children, j in vaccine)
        {
        sum(l in day) x[i][j][l]*l == t[i][j];
        }

    forall (i in children, j in vaccine, k in day)
    {
        u[i][j] == t[i][j]*w[i][j];}

    forall (i in children, l in day)
    {
        sum(j in vaccine) x[i][j][l] <= 2;}

    forall (i in children, j in vaccine)
    {
        sum(l in day) x[i][j][l] == 1;}}
```

References

Table 1: Scheduling Charts 1 to 5

Day Number Run	First 5 Scheduling Runs (Result & Cumulative After Each Run)								
	Run 1 Result	Run 2 Result	Cum.	Run 3 Result	Cum.	Run 4 Result	Cum.	Run 5 Result	Cum.
28	2	-	-	-	-	-	-	-	-
29	2	0	2	-	-	-	-	-	-
30	1	1	2	0	2	-	-	-	-
31	1	1	2	1	3	1	4	-	-
32	1	1	2	1	3	1	4	1	5
33	2	0	2	1	3	0	3	1	4
34	1	1	2	1	3	0	3	1	4
35	1	1	2	0	2	1	3	1	4
36	1	1	2	0	2	1	3	2	5
37	1	0	1	1	2	1	3	1	4
38	1	1	2	0	2	1	3	1	4
39	1	0	1	1	2	0	2	1	3
40	1	1	2	0	2	1	3	1	4
41	1	1	2	0	2	0	2	1	3
42	1	1	2	0	2	1	3	1	4
43	1	0	1	1	2	1	3	1	4
44	1	1	2	0	2	0	2	2	4
45	1	1	2	0	2	1	3	1	4
46	1	1	2	0	2	0	2	2	4
47	1	0	1	1	2	1	3	1	4
48	1	1	2	0	2	1	3	1	4
49	1	0	1	1	2	1	3	1	4
50	1	0	1	1	2	1	3	2	5
51	1	0	1	1	2	0	2	1	3
52	2	0	2	0	2	1	3	1	4
53	1	1	2	0	2	0	2	1	3
54	1	1	2	1	3	0	3	1	4
55	1	1	2	0	2	1	3	1	4
56	2	0	2	0	2	1	3	0	3
57	1	1	2	0	2	0	2	1	3
58	1	0	1	1	2	1	3	1	4
59	1	1	2	1	3	0	3	1	4
60	1	0	1	1	2	1	3	0	3
61	-	1	1	1	2	0	2	2	4
62	-	-	-	2	2	1	3	1	4
63	-	-	-	-	-	2	2	1	3
64	-	-	-	-	-	-	-	4	4

Table 2: Scheduling Charts 11 to 15

Day Number Run	11th-15th Scheduling Runs (Result & Cumulative After Each Run)									
	Run 11 Result	Cum.	Run 12 Result	Cum.	Run 13 Result	Cum.	Run 14 Result	Cum.	Run 15 Result	Cum.
38	0	7	-	-	-	-	-	-	-	-
39	1	8	0	8	-	-	-	-	-	-
40	0	7	1	8	1	9	-	-	-	-
41	0	8	1	9	0	9	1	10	-	-
42	0	8	1	9	0	9	0	9	1	10
43	1	8	1	9	0	9	1	10	0	10
44	1	8	0	8	1	9	0	9	1	10
45	0	8	1	9	0	9	0	9	1	10
46	0	7	1	8	1	9	0	9	0	9
47	1	8	0	8	1	9	1	10	0	10
48	0	7	1	8	0	8	1	9	1	10
49	0	8	0	8	0	8	1	9	1	10
50	1	8	0	8	0	8	1	9	0	9
51	0	7	1	8	0	8	1	9	0	9
52	0	8	0	8	0	8	0	8	2	10
53	0	7	1	8	0	8	0	8	1	9
54	1	8	0	8	0	8	0	8	1	9
55	0	7	0	7	1	8	1	9	0	9
56	1	8	0	8	0	8	0	8	1	9
57	0	7	1	8	0	8	1	9	0	9
58	1	9	0	9	0	9	0	9	0	9
59	0	7	1	8	0	8	0	8	1	9
60	1	8	0	8	0	8	1	9	0	9
61	0	8	0	8	0	8	0	8	1	9
62	1	8	0	8	0	8	0	8	1	9
63	0	7	0	7	1	8	1	9	0	9
64	1	8	0	8	0	8	0	8	1	9
65	1	8	0	8	0	8	0	8	1	9
66	1	8	0	8	0	8	1	9	0	9
67	1	8	1	9	0	9	0	9	0	9
68	1	8	0	8	0	8	0	8	0	8
69	1	8	0	8	0	8	1	9	0	9
70	7	7	1	8	0	8	0	8	1	9
71	-	-	6	6	1	7	1	8	1	9
72	-	-	-	-	7	7	0	7	1	8
73	-	-	-	-	-	-	6	6	2	8
74	-	-	-	-	-	-	-	-	7	7

Table 3: Scheduling Charts 21 to 25

Day Number Run	21st-25th Scheduling Runs (Result & Cumulative After Each Run)									
	Run 21 Result	Cum.	Run 22 Result	Cum.	Run 23 Result	Cum.	Run 24 Result	Cum.	Run 25 Result	Cum.
48	1	13	-	-	-	-	-	-	-	-
49	0	12	1	13	-	-	-	-	-	-
50	0	13	0	13	0	13	-	-	-	-
51	1	13	0	13	0	13	1	14	-	-
52	0	12	1	13	0	13	1	14	0	14
53	1	13	0	13	1	14	0	14	0	14
54	1	12	1	13	0	13	0	13	1	14
55	1	13	0	13	1	14	0	14	0	14
56	2	13	0	13	0	13	0	13	1	14
57	0	12	1	13	0	13	0	13	0	13
58	1	13	0	13	1	14	0	14	0	14
59	1	13	0	13	1	14	0	14	0	14
60	0	12	0	12	1	13	0	13	0	13
61	1	13	1	14	1	15	0	15	0	15
62	0	12	1	13	1	14	0	14	0	14
63	1	13	0	13	0	13	1	14	0	14
64	0	12	1	13	1	14	0	14	0	14
65	1	12	0	12	1	13	0	13	1	14
66	0	12	1	13	0	13	0	13	1	14
67	1	12	0	12	1	13	1	14	1	15
68	1	12	1	13	0	13	1	14	0	14
69	0	12	0	12	1	13	1	14	0	14
70	1	12	0	12	1	13	1	14	1	15
71	0	12	0	12	1	13	0	13	0	13
72	0	11	1	12	1	13	0	13	0	13
73	0	11	0	11	2	13	1	14	0	14
74	1	12	0	12	1	13	0	13	1	14
75	0	10	0	10	1	11	2	13	0	13
76	0	10	0	10	1	11	1	12	0	12
77	0	11	0	11	0	11	2	13	0	13
78	0	10	0	10	1	11	0	11	1	12
79	0	10	0	10	0	10	1	11	0	11
80	8	8	1	9	1	10	0	10	0	10
81	-	-	0	7	1	8	1	9	0	9
82	-	-	-	-	6	6	1	7	0	7
83	-	-	-	-	-	-	6	6	1	7
84	-	-	-	-	-	-	-	-	7	7

Table 4: Scheduling Charts 31 to 35

Day Number Run	31st-35th Scheduling Runs (Result & Cumulative After Each Run)									
	Run 31 Result	Cum.	Run 32 Result	Cum.	Run 33 Result	Cum.	Run 34 Result	Cum.	Run 35 Result	Cum.
57	0	16	-	-	-	-	-	-	-	-
58	0	15	1	16	-	-	-	-	-	-
59	0	16	1	17	0	18	-	-	-	-
60	0	16	0	16	1	17	0	17	-	-
61	0	15	1	16	0	16	1	17	0	17
62	0	15	1	16	1	17	0	17	0	17
63	0	15	1	16	0	16	0	16	1	17
64	0	15	1	16	1	17	0	17	0	17
65	0	15	1	16	0	16	0	16	0	16
66	0	16	0	16	1	17	0	17	0	17
67	0	15	0	15	2	17	0	17	0	17
68	1	16	0	16	0	16	0	16	1	17
69	0	15	0	15	0	15	0	15	2	17
70	0	15	1	16	0	16	0	16	0	16
71	0	15	0	15	1	16	0	16	1	17
72	0	15	0	15	0	15	0	15	2	17
73	1	16	0	16	1	17	0	17	0	17
74	0	15	1	16	1	17	0	17	0	17
75	0	14	0	14	0	14	0	14	2	16
76	1	15	1	16	0	16	0	16	0	16
77	0	14	2	16	0	16	1	17	0	17
78	1	14	0	14	2	16	0	16	1	17
79	0	12	2	14	2	16	0	16	0	16
80	0	12	2	14	2	16	0	16	1	17
81	1	14	1	15	0	15	1	16	0	16
82	2	14	1	15	0	15	0	15	1	16
83	0	13	1	14	2	16	0	16	0	16
84	1	13	0	13	2	15	1	16	0	16
85	0	13	0	13	1	14	2	16	1	17
86	0	13	0	13	1	14	1	15	1	16
87	0	12	0	12	1	13	2	15	0	15
88	0	10	0	10	2	12	2	14	0	14
89	9	9	1	10	2	12	2	14	0	14
90	-	-	8	8	2	10	3	13	1	14
91	-	-	-	-	9	9	4	13	1	14
92	-	-	-	-	-	-	9	9	2	11
93	-	-	-	-	-	-	-	-	8	8

Table 5: Scheduling Charts 41 to 45

Day Number Run	41st-45th Scheduling Runs (Result & Cumulative After Each Run)									
	Run 41 Result	Cum.	Run 42 Result	Cum.	Run 43 Result	Cum.	Run 44 Result	Cum.	Run 45 Result	Cum.
67	0	17	-	-	-	-	-	-	-	-
68	0	18	0	18	-	-	-	-	-	-
69	0	18	0	18	0	18	-	-	-	-
70	1	18	0	18	0	18	0	18	-	-
71	0	17	0	17	0	17	0	17	0	17
72	0	18	1	19	0	19	0	19	0	19
73	0	19	0	19	0	19	0	19	0	19
74	0	17	0	17	0	17	0	17	0	17
75	0	18	0	18	0	18	0	18	0	18
76	0	19	0	19	0	19	0	19	0	19
77	0	18	0	18	1	19	0	19	0	19
78	0	17	1	18	0	18	0	18	0	18
79	0	19	0	19	0	19	0	19	0	19
80	0	18	0	18	0	18	0	18	0	18
81	1	17	0	17	0	17	0	17	1	18
82	0	18	0	18	0	18	0	18	0	18
83	1	18	0	18	0	18	0	18	0	18
84	0	18	0	18	1	19	0	19	0	19
85	0	17	0	17	0	17	0	17	0	17
86	0	18	0	18	0	18	0	18	0	18
87	0	19	0	19	0	19	0	19	0	19
88	1	17	0	17	0	17	0	17	1	18
89	0	18	0	18	0	18	0	18	0	18
90	1	19	0	19	0	19	0	19	0	19
91	0	17	0	17	0	17	1	18	0	18
92	0	17	0	17	0	17	1	18	0	18
93	0	18	1	19	0	19	0	19	0	19
94	0	15	2	17	0	17	0	17	0	17
95	0	13	2	15	0	15	2	17	1	18
96	0	10	1	11	0	11	4	15	1	16
97	1	13	1	14	0	14	2	16	1	17
98	2	12	1	13	0	13	1	14	0	14
99	9	9	2	11	0	11	0	11	0	11
100	-	-	9	9	2	11	1	12	0	12
101	-	-	-	-	10	10	0	10	0	10
102	-	-	-	-	-	-	9	9	2	11
103	-	-	-	-	-	-	-	-	8	8
